# Evaluation of *Phaseolus vulgaris* Extract in a Rat Model of Cafeteria-Diet-Induced Obesity: Metabolic and Biochemical Effects

**DOI:** 10.3390/foods14122038

**Published:** 2025-06-09

**Authors:** Atcha Uawongwattana, Kakanang Posridee, Kittipong Promyo, Atcharaporn Thaeomor, Ratchadaporn Oonsivilai

**Affiliations:** 1 School of Food Technology, Institute of Agricultural Technology, Suranaree University of Technology, Nakhon Ratchasima 30000, Thailand; atcha8@gmail.com (A.U.); posridee.ka@gmail.com (K.P.); kittipong.p83@sut.ac.th (K.P.); 2 School of Preclinical Science, Institute of Science, Suranaree University of Technology, Nakhon Ratchasima 30000, Thailand; 3 Health and Wellness Research Unit, Institute of Research and Development, Suranaree University of Technology, Nakhon Ratchasima 30000, Thailand

**Keywords:** obesity, cafeteria diet, *Phaseolus vulgaris*, glucose tolerance test, blood chemistry

## Abstract

Obesity is a global health concern that elevates the risk of noncommunicable diseases (NCDs) such as type 2 diabetes, cardiovascular disease, and certain cancers. *Phaseolus vulgaris* (white bean) contains α-amylase inhibitors (αAIs) that can reduce carbohydrate digestion and absorption, potentially mitigating obesity and metabolic syndrome. This study investigated the impact of *P. vulgaris* extract (PVE) on obese rats. Male Wistar rats were fed either a standard diet (SD) or a cafeteria diet (CAF) for 17 weeks to induce obesity. Subsequently, rats in each dietary group were randomly assigned to receive a vehicle, low-dose PVE (200 mg/kg), high-dose PVE (300 mg/kg), or metformin (200 mg/kg) via an oral gavage for 6 weeks. The CAF group exhibited significantly greater weight gain compared to the SD group. In the CAF group, a low dose of PVE lowered postprandial glycemia during an oral glucose tolerance test (OGTT) at 60 and 120 min and decreased food and energy intake during weeks 17–20 and 18–19, respectively. In the SD group, a high dose of PVE reduced glycemia at 90 min in the OGTT, as well as body weight gain, food intake, and energy intake during week 17. However, the overall areas under the glucose curves in the OGTT were not significantly different across treatment groups (*p* > 0.05), and while individual time points showed changes, the overall glucose exposure (AUC) was not significantly altered. In conclusion, the αAIs present in *P. vulgaris* demonstrate the potential to reduce body weight, weight gain, glycemia, total cholesterol, and triglycerides in vivo, but in the CAF group, neither PVE dose significantly altered the TC or TG. This study provides strong support for further exploring *Phaseolus vulgaris* extract as a valuable functional ingredient in the food industry, particularly for developing products that aid in weight management and glycemic control.

## 1. Introduction

Noncommunicable chronic diseases (NCDs), including hypertension, hyperlipidemia, type 2 diabetes mellitus (T2DM), and metabolic syndrome, are leading causes of global mortality [1]. Obesity and excessive weight are significant modifiable risk factors for NCDs, which are primarily driven by dietary patterns characterized by an imbalance between energy intake and expenditure [2,3]. T2DM, a prevalent NCD, is characterized by impaired insulin action and hyperglycemia and is often associated with obesity and lifestyle factors [4].

Dietary bioactive compounds offer a potential preventative and therapeutic strategy against these conditions, among which α-amylase inhibitors (αAIs), notably found in white kidney beans (Phaseolus vulgaris), can retard starch absorption. First identified in 1945 [5] and later termed phaseolamin [6], αAIs from *P. vulgaris* have been extensively investigated for their effects on T2DM, excessive weight, and obesity in both animal models [7,8,9,10,11] and human subjects [12,13,14]. *P. vulgaris* extract (PVE) has been reported to reduce body weight and improve glycemic control in various animal models [7,8,10,15]. In addition, a previous study by Oonsivilai et al. (2024) [16] successfully employed a randomized Box–Behnken design to identify the optimal conditions for obtaining white kidney bean extract with maximized yield and alpha-amylase inhibitory activity. Under these parameters, the extract achieved a promising yield of 56.75% and a notable inhibitory activity of 2.85 units/g.

Collectively, studies on *Phaseolus vulgaris* extract (PVE) and its active compound, phaseolamin, demonstrate significant potential for managing metabolic disorders in animal models. The core mechanism involves α-amylase inhibition, which delays carbohydrate digestion and absorption, leading to reduced post-prandial glucose levels and contributing to hypoglycemic and anorexigenic effects [7,8,10]. This translates into beneficial outcomes such as decreased food intake, reduced body weight, and improved glycemic control [7,8,10]. Beyond these direct metabolic impacts, PVE has also shown protective effects against oxidative stress and organ damage associated with diabetes and metabolic syndrome, including the prevention of collagen deposition in the heart and a reduction in hepatic steatosis [9,10]. While the overall evidence supports PVE’s efficacy, some inconsistencies regarding dose–response relationships [9] highlight the need for further research to fully optimize its therapeutic application in various physiological contexts.

Despite the recognized potential of *P. vulgaris* components, the precise relationship between extract dosage and metabolic outcomes remains inconsistent. For instance, some studies have reported that varying doses of phaseolamin uniformly reduced glycemia without demonstrating a clear dose-dependent response, and in certain induced diabetes models, phaseolamin did not significantly alter key metabolic markers [9]. While other research has shown PVE to be comparable to established antidiabetic drugs like metformin in improving glycemic profiles [10], these contrasting findings underscore a critical need for further investigation into the specific dose–response mechanisms and the broader impact of PVE on diverse metabolic parameters.

The cafeteria diet (CAF)-induced obese rat model is a widely used research tool that mimics human obesity by providing rats with ad libitum access to a variety of highly palatable, energy-dense “junk foods” in addition to their standard chow. This encourages voluntary hyperphagia (overeating) due to the appeal and diversity of the foods, leading to rapid weight gain and increased adiposity. Beyond simple weight gain, the model consistently induces a comprehensive metabolic syndrome phenotype, including hyperinsulinemia, insulin resistance, dyslipidemia, and nonalcoholic fatty liver disease, closely reflecting the complex metabolic dysfunctions seen in humans with diet-induced obesity. This approach leverages the rats’ natural preferences to create a robust and translationally relevant model for studying obesity and its comorbidities.

A significant research gap exists in evaluating the therapeutic efficacy of *P. vulgaris* extracts in established diet-induced obesity models, particularly those induced by a prolonged cafeteria diet (CAF). Much of the existing literature has focused on the preventative or early ameliorative effects of P. vulgaris administration, often initiating treatment concurrently with or shortly after obesogenic diet exposure. To address these limitations and provide clinically relevant insights, this study investigated the therapeutic potential of *P. vulgaris* extract (PVE) in Wistar rats with well-established obesity induced by a prolonged CAF regimen. This approach facilitates a comprehensive assessment of PVE’s capacity to reverse or mitigate the metabolic consequences of chronic diet-induced obesity, specifically focusing on glycemia and key blood biochemical parameters, thereby distinguishing this study from prior research and offering novel insights into PVE as an intervention for existing metabolic complications.

## 2. Materials and Methods

### 2.1. Materials and Sample Preparation

The extraction of αAIs from *P. vulgaris* was optimized based on established protocols [8,10]. In brief, 1.65 g of finely ground white kidney bean powder was suspended in 10 mL of 0.1 M phosphate-buffered saline (PBS, pH 7.2 containing 150 mM NaCl), resulting in a 1:6.06 (*w*/*v*) ratio. This suspension was continuously stirred at 37 °C for 1 h to facilitate the release of inhibitory compounds. Following incubation, the homogenate was centrifuged at 10,000 rpm (equivalent to approximately 16,000× *g*) for 30 min at 4 °C using a refrigerated centrifuge (Hettich, Herford, Germany, universal 16R, USA). The resulting supernatant, containing the crude PVE, was carefully collected and adjusted to a final volume of 10 mL with the PBS solution. Aliquots of 2 mL were then prepared and immediately freeze-dried using a freezer (GEA, LYOVAC GT2-S, MD, Hurth, Germany) at −80 °C under a pressure of 0.010 mBar for 72 h. The freeze-dried PVE aliquots were stored at −20 °C until further biochemical analysis. The protein content of the reconstituted PVE was determined using the Bradford assay with bovine serum albumin (BSA) as a standard [15]. For the Bradford assay, first prepare a BSA solution at a concentration of 1 mg/mL and dilute it to concentrations of 0, 0.2, 0.4, 0.6, 0.8, and 1.0 mg/mL to establish a standard curve. Prepare the Bradford reagent by dissolving 100 mL of Coomassie Brilliant Blue G-250 in 50 mL of 95% ethanol, stirring until the dye is completely dissolved. Next, add 100 mL of 85% phosphoric acid, and adjust the final volume to 1000 mL with distilled water. Filter the solution using filter paper No. 1 and store it in an amber bottle at 4 °C. For protein measurement, mix 25 µL of the sample or standard solution with 1 mL of the Bradford reagent. Incubate the solution at room temperature for 5 min; then, measure the absorbance using a spectrophotometer (Genesys 10S UV-VIS, Thermo Fisher Scientific, Waltham, MA, USA) at a wavelength of 595 nm.

### 2.2. SDS PAGE

The separating gel was poured into the gel forming stand, covered with methanol, and waited around 30 min. The methanol was rinsed out, The taging gel was poured and quickly inserted with the comb, waited around 10 min stored in -4°C until use. The calculated sample solution, for loading 20 µL protein in each well of the gel, was mixed with the 2X sample buffer containing 2.5% mercaptoethanol in ratio 1:1, heated at 95 °C for 5 min. The tank compound with gel holder and gel cassette was builded up, the running buffer was poured in and the comb was carefully removed. The marker protein and mixed sample was loaded into the gel well. Cover the tank with the tank lid and connect the electrode to the power supplier. The power supplier was set with 150 volts, and 60 min running time and run. After the run was complete, the gel was extracted, the staging gel part was cut off, the gel was washed with tap water, stained with Coomassie blue solution with shaking for 4 h. After staining was completed, the gel was destained with tap water with shaking for overnight. The gel was stored at room temperature until photographing.

### 2.3. Animals and Experimental Design

#### 2.3.1. Animal Experiment for Obesity Induction Using SD and CAF

Sixty-four male Wistar rats, aged 8 weeks upon arrival, were obtained from the Laboratory Animal Center at Suranaree University of Technology (SUT), Nakhon Ratchasima, Thailand. Upon arrival, rats were housed individually in standard plastic cages under controlled environmental conditions, maintaining a constant temperature (22–24 °C) and a 12 h light/dark cycle. All experimental procedures adhered strictly to the ethical guidelines for the care and use of laboratory animals established by the Suranaree University of Technology Animal Care and Use Committee, and the study was conducted under the approved protocol (approval code A-12/2020).

Following a two-week acclimation period, the rats were randomly assigned to one of two dietary groups (*n* = 24 per group): a standard rodent chow diet (SD) group or a cafeteria diet (CAF) group. The CAF, designed to induce obesity, consisted of a variety of palatable, high-energy foods offered in addition to standard chow for a duration of 17 weeks, as detailed in the Supplementary Material.

#### 2.3.2. Animal Experiment During Treatment

After the 17-week dietary intervention, both the SD and CAF groups were further randomized into four treatment subgroups (*n* = 6 per subgroup) for a subsequent 6-week treatment period. The SD group comprised a vehicle control (SDV; distilled water), a low-dose PVE group (SDLP; 200 mg/kg body weight), a high-dose PVE group (SDHP; 300 mg/kg body weight), and a metformin-treated group (SDM; 200 mg/kg body weight). Similarly, the CAF group included a vehicle control (CAFV; distilled water), a low-dose PVE group (CAFLP; 200 mg/kg body weight), a high-dose PVE group (CAFHP; 300 mg/kg body weight), and a metformin-treated group (CAFM; 200 mg/kg body weight). All treatments were administered orally via a gavage once daily throughout the 6-week treatment duration. In addition, the low and high doses of PVE were chosen based on αAI activities determined during the extract preparation process in previous research [7,8,9,10].

All treatments were administered orally via a gavage once daily for 6 weeks. At the end of the 6-week treatment period, all rats underwent an oral glucose tolerance test (OGTT). Following the OGTT, rats were fasted overnight (12 h) before being humanely sacrificed by CO_2_ asphyxiation and subsequently euthanized via cardiac puncture for blood sample collection. Blood samples were processed to obtain serum and plasma for subsequent biochemical analyses.

### 2.4. Oral Glucose Tolerance Test

An oral glucose tolerance test (OGTT) was performed after 6 weeks of oral administration. All rats in the groups were given glucose (2 g/kg) after 12 h fasting. These measurements were taken at 0 (before oral glucose), 30, 60, 90, and 120 min after oral glucose administration at 2 g/kg BW, and the area under the curve (AUC) was determined by calculation of the trapezoid area formula and accumulation [17,18].

### 2.5. Blood Chemistry

Following the experimental period, rat blood collected in anticoagulant-treated tubes was centrifuged at 10,000 rpm at 4 °C for 5 min. Plasma was then carefully separated, aliquoted, and stored at −20 °C pending an analysis performed by a certified laboratory service (RIA Laboratory Co., Ltd., Nakhon Ratchasima, Thailand) with the standard methods certified by Laboratory Thailand accreditation by Thailand Medical Technology Council. The blood chemistry profile assessed included the quantification of total cholesterol (TC), triglyceride (TG) levels, low-density lipoprotein (LDL), high-density lipoprotein (HDL), aspartate transaminase (AST), alanine transaminase (ALT), blood urea nitrogen (BUN), and creatinine (CR).

### 2.6. Histopathological Analysis

After the experimental period, rats were euthanized, and target organs, such as the liver, were carefully removed and immediately placed in 10% neutral buffered formalin. Once adequately fixed, these tissue samples underwent routine histological preparation for microscopic analysis. This involved a dehydration series using ascending concentrations of ethanol (50%, 60%, 70%, 80%, 95%, and absolute ethanol [*v*/*v*]), followed by clearing in xylene. Subsequently, the cleared tissues were infiltrated with molten paraffin wax and embedded into cassettes. Sections of approximately 4 µm thickness were cut from the paraffin blocks using a rotary microtome. These sections were then floated on a 50 °C water bath, collected onto glass microscope slides, and dried in a 70 °C oven. Prior to staining, sections were deparaffinized in xylene and rehydrated through a descending ethanol series (100%, 95%) before rinsing in tap water. Hematoxylin and eosin (H&E) staining was performed, involving hematoxylin application, tap water rinse, and eosin counterstain. Post-staining, sections were dehydrated through graded ethanol (95%, 100%), cleared in xylene, and permanently mounted. Finally, the prepared slides were examined under a light microscope to conduct histopathological assessments.

### 2.7. Statistical Analysis

All experiments were performed in biological replicates, and mean values (on a dry basis) with standard deviations are reported. Data were analyzed via an independent-sample T-test with a statistically significant difference at *p* < 0.05. The experimental data were analyzed using an analysis of variance (ANOVA). The software platform SPSS^®^ version 17 (SPSS Inc., Chicago, IL, USA) was used to perform all statistical calculations.

## 3. Results

### 3.1. SDS PAGE

**Figure 1:**
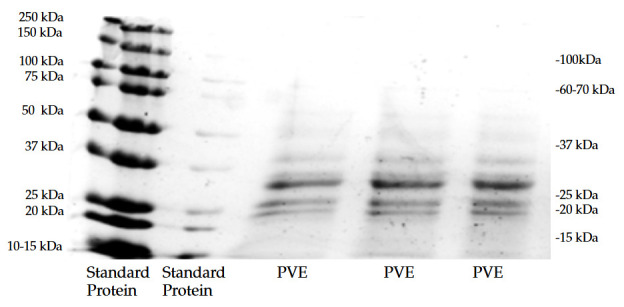
SDS PAGE of PVE.

The result of sodium dodecyl sulfate-polyacrylamide gel electrophoresis (SDS-PAGE) is shown in Figure 1. The PVE samples analyzed via SDS-PAGE exhibited a broad distribution of protein molecular weights, ranging from very high molecular weight components (likely above 250 kDa—possibly indicative of aggregates or large protein complexes) down to smaller proteins around 100 kDa, 60–70 kDa, 37 kDa, 25 kDa, and even as low as 20 kDa and possibly 15 kDa. Marshall and Lauda (1975) [6] reported their phaseolamin molecular weight in the range 45,000–50,000 kDa. Pueyo et al. (1993) reported their αAI small polypeptide molecular weight 14,000–18,000 kDa [19].

### 3.2. Body Weight

The initial body weights did not differ significantly between the standard diet (SD) or cafeteria diet (CAF) groups during the first two weeks (*p* > 0.05; Figure 1). Subsequently, from week 3 to week 16 (prior to subgroup allocation), the CAF group exhibited significantly higher body weights compared to the SD group (*p* < 0.05). Following subgrouping at week 17, rats in the CAF group maintained significantly higher body weights than all the SD subgroups receiving the same oral administration (*p* < 0.05), except for the metformin-treated subgroups at week 18 (*p* > 0.05). Within the SD group, no significant differences in body weight were observed between the vehicle control and the low-PVE-, high-PVE-, and metformin-treated subgroups in the weeks following oral administration (*p* > 0.05), except for the high-PVE subgroup at the final week (week 22), which displayed a significantly lower body weight compared to the vehicle control (*p* < 0.05). Similarly, within the CAF group, body weights did not significantly differ between the vehicle control and the low-PVE- and high-PVE-treated subgroups after oral administration (*p* > 0.05), except for the metformin-treated subgroup at week 18 and the final week, which showed significantly lower body weights compared to the vehicle control (*p* < 0.05).

The initial body weights were comparable across all SD and CAF subgroups receiving the same oral administration (*p* > 0.05) (Table 1). Furthermore, within both the SD and CAF groups, the initial body weights did not significantly differ between the vehicle control and the subgroups treated with low PVE, high PVE, or metformin (*p* > 0.05). At the conclusion of the study, the CAF group exhibited significantly higher final body weights and total body weight gain compared to the corresponding SD subgroups receiving the same oral administration (*p* < 0.05). Within the SD group, high-PVE administration resulted in a significantly lower final body weight and body weight gain compared to the vehicle control (*p* < 0.05). Low-PVE administration in the SD group led to a significant reduction in body weight gain compared to the vehicle control (*p* < 0.05), although the final body weight did not differ significantly (*p* > 0.05), while metformin administration in the SD group did not significantly affect the final body weight or body weight gain compared to the vehicle control (*p* > 0.05). Conversely, within the CAF group, metformin administration significantly reduced both the final body weight and body weight gain compared to the vehicle control (*p* < 0.05). Low-PVE administration in the CAF group resulted in a significantly lower body weight gain compared to the vehicle control (*p* < 0.05), but the final body weight was not significantly different (*p* > 0.05), while high-PVE administration in the CAF group did not significantly alter the final body weight or body weight gain compared to the vehicle control (*p* > 0.05).

### 3.3. Food Intake

Food intake was monitored weekly (Table 2). Prior to subgrouping, no significant differences in food intake were observed between the SD and CAF groups during the initial two weeks or at week 6 (*p* > 0.05). However, from weeks 3 to 5 and weeks 7 to 16, the CAF group consumed significantly less food than the SD group (*p* < 0.05). Following subgrouping, in the vehicle-treated subgroups, the CAF group exhibited a comparable food intake to the SD group, except at weeks 17 and 20, where the CAF group showed significantly lower consumption (*p* < 0.05). In the low-PVE-treated subgroups, the CAF group consistently displayed significantly lower food intake than the SD group throughout the post-subgrouping period (*p* < 0.05). In the high-PVE-treated subgroups, food intake did not significantly differ between the CAF and SD groups, except in weeks 17 and 21, where the CAF group consumed less (*p* < 0.05). Similarly, in the metformin-treated subgroups, the CAF group consistently exhibited a significantly lower food intake compared to the SD group after subgrouping (*p* < 0.05).

Within the SD group, the food intake did not significantly differ across the orally administered subgroups (vehicle, low PVE, high PVE, and metformin) during the treatment period (*p* > 0.05), except for week 17 for the low-PVE, high-PVE, and metformin subgroups and week 18 for the metformin subgroup, where the food intake was significantly lower than that of the vehicle control (*p* < 0.05). Among the CAF group, the low-PVE subgroup exhibited a significantly lower food intake compared to the vehicle control from weeks 17 to 20 (*p* < 0.05), and the metformin subgroup showed significantly reduced food intake from weeks 17 to 22 (*p* < 0.05). In contrast, the low-PVE subgroup from week 21 to 22 and the high-PVE subgroup from week 17 to 22 did not show significant differences in the food intake compared to the vehicle control within the CAF group (*p* > 0.05). Notably, the food intake in the SD group remained relatively stable from week 4 until the end of the experiment, even after subgrouping. Similarly, the CAF group’s food intake remained consistent from week 3 until the study’s conclusion despite the subgroup allocation.

### 3.4. Energy Intake

The energy intake was calculated based on weekly food consumption (Table 3). During the initial 16 weeks (excluding week 4), the CAF group exhibited a significantly higher energy intake compared to the SD group (*p* < 0.05). During the oral administration period (weeks 17–22), the vehicle-treated CAF and SD groups did not significantly differ in energy intake (*p* > 0.05). However, for the low-PVE-, high-PVE-, and metformin-treated subgroups, the energy intake was comparable between the SD and CAF groups at week 17 (*p* > 0.05), but the CAF subgroups showed a significantly higher energy intake from weeks 18 to 22 compared to their respective SD counterparts (*p* < 0.05). Within the SD group, the energy intake remained consistent across all the treatment subgroups (vehicle, low PVE, high PVE, and metformin) after subgrouping (*p* > 0.05) except for week 17, where the low- and high-PVE subgroups exhibited significantly lower energy intakes than the vehicle control (*p* < 0.05), and weeks 17 and 18, where the metformin subgroup showed a significantly higher energy intake than the vehicle control (*p* < 0.05). Within the CAF group, the energy intake did not significantly differ across treatment subgroups after subgrouping (*p* > 0.05), except for the low-PVE subgroup at weeks 18 and 19, which showed a significantly lower energy intake than the vehicle control (*p* < 0.05). The SD group maintained a stable energy intake trend throughout the experiment, even after subgrouping. In contrast, the CAF group displayed a notably high energy intake in the first week, followed by a consistent trend from week 2 onwards, which was unaffected by the subgroup allocation.

### 3.5. Oral Glucose Tolerance Test (OGTT)

The oral glucose tolerance test (OGTT) results are presented in Table 4. In the control group (CAF) receiving the vehicle, the fasting blood glucose levels (0 min) and glucose concentrations at 30, 60, 90, and 120 min post-glucose administration were significantly elevated compared to the standard diet (SD) group (*p* < 0.05). Following oral administration of both low and high doses of PVE, the CAF group exhibited significantly higher blood glucose levels at 30, 60, 90, and 120 min compared to the SD group receiving the same PVE dose (*p* < 0.05). Similarly, the CAF group treated with metformin showed significantly higher blood glucose concentrations at 60, 90, and 120 min post-glucose load compared to the SD group administered metformin (*p* < 0.05).

Within the SD group, no significant differences were observed in the fasting blood glucose or glucose levels at 30, 60, and 120 min following the administration of low PVE, high PVE, or metformin compared to the vehicle control (*p* > 0.05). An exception was noted at the 90 min time point, where the high-PVE-treated SD group displayed significantly higher blood glucose levels than the vehicle control (*p* < 0.05). Among the CAF groups, no significant differences were found in fasting blood glucose or blood glucose concentrations across all time points following the administration of low PVE, high PVE, or metformin compared to the vehicle control (*p* > 0.05). However, the CAF group receiving low PVE exhibited significantly lower blood glucose levels at 60, 90, and 120 min, and the CAF group receiving metformin showed significantly lower blood glucose at 120 min compared to the CAF vehicle control (*p* < 0.05).

The area under the curve (AUC) for the OGTT is depicted in Table 5. Despite a nonsignificant trend toward higher AUC values in the CAF groups, no statistically significant differences in AUC were found between the SD and CAF groups across all treatments (*p* > 0.05). Furthermore, within each diet group (SD and CAF), no significant differences in the AUC were found between the vehicle control and the groups treated with low PVE, high PVE, or metformin (*p* > 0.05). Notably, the CAF group treated with low PVE (CAFLP) showed a slight decrease in the AUC compared to other groups within the same diet.

### 3.6. Blood Chemistry

All groups exhibited consistent but incomparable LDL levels (Table 6). When administered orally, there were no statistically significant differences in total cholesterol (TC), triglyceride (TG) levels, or high-density lipoprotein (HDL) between the SD and CAF groups (*p* > 0.05), with one exception: The CAF group receiving low PVE demonstrated significantly higher TG levels compared to the SD group under the same low-PVE administration (*p* < 0.05).

Within the SD group, both the low- and high-PVE administration resulted in significantly lower TC levels compared to the vehicle control (*p* < 0.05), while the metformin treatment showed no significant difference from the vehicle. Conversely, within the CAF groups, neither the low-PVE, high-PVE, nor metformin administration significantly altered the TC levels compared to the vehicle control (*p* > 0.05).

Regarding the TG levels in the SD group, only the low-PVE administration led to a significant reduction compared to the vehicle control (*p* < 0.05). The high-PVE and metformin treatments in the SD group did not significantly differ from the vehicle control (*p* > 0.05). In contrast, no significant differences in the TG levels were observed between the low-PVE-, high-PVE-, or metformin-treated CAF groups and the vehicle control (*p* > 0.05).

Both the low- and high-PVE administration resulted in significantly lower HDL levels in the SD group compared to the vehicle control (*p* < 0.05). Metformin treatment in the SD group did not significantly affect the HDL levels compared to the vehicle control (*p* < 0.05). Similarly, no significant differences in the HDL levels were found between the low-PVE-, high-PVE-, or metformin-treated CAF groups and the vehicle control (*p* > 0.05).

No statistically significant differences in aspartate aminotransferase (AST) or alanine aminotransferase (ALT) levels were observed between the SD and CAF groups under the same oral administration (*p* > 0.05), with two exceptions: Firstly, the CAF group administered the vehicle control exhibited significantly lower AST levels compared to the SD group (*p* < 0.05). Secondly, the CAF group treated with metformin showed significantly lower ALT levels compared to the SD group (*p* < 0.05).

More interestingly, within the SD group, both the low- and high-PVE administration resulted in significantly lower AST and ALT levels compared to the vehicle control (*p* < 0.05). In contrast, in the SD group, metformin treatment did not significantly alter the AST or ALT levels compared to the vehicle control (*p* > 0.05). Similarly, in the CAF groups, neither low-PVE, high-PVE, nor metformin administration significantly affected the AST or ALT levels compared to the vehicle control (*p* > 0.05).

### 3.7. Histology

**Figure 2:**
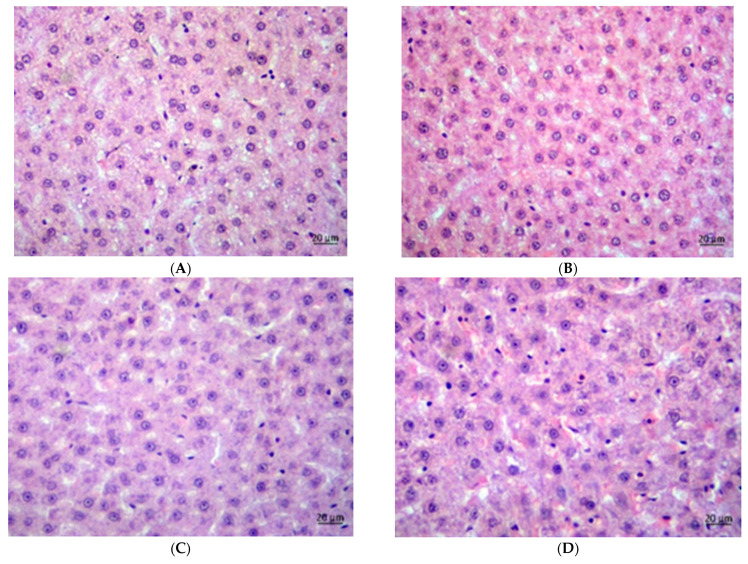
Histology of livers of male rats received: SDV (**A**), SDLP (**B**), SDHP (**C**), SDM (**D**).

**Figure 3:**
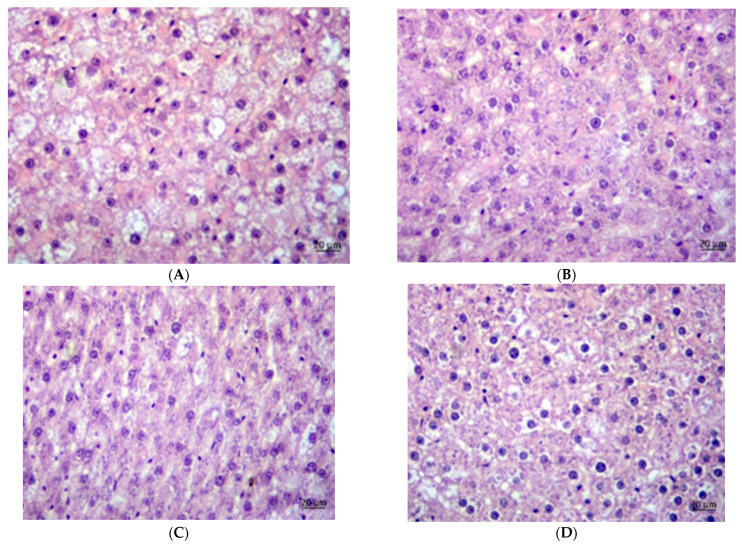
Histology of livers of male rats received: CAFV (**A**), CAFLP (**B**), CAFHP (**C**), CAFM (**D**).

**Figure 4:**
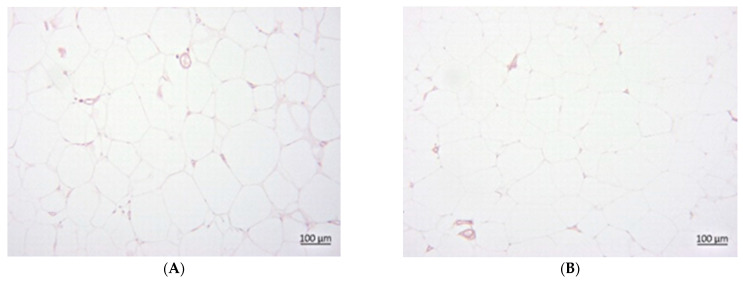

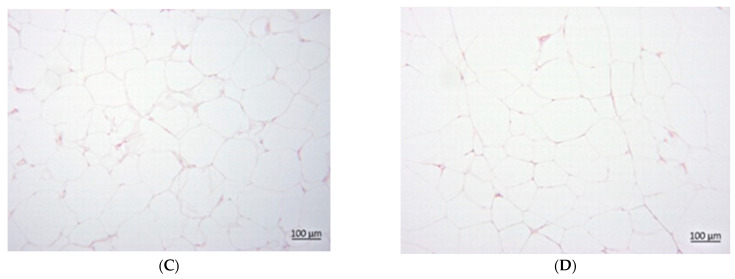
eWAT of SDV (**A**), SDLP (**B**), SDHP (**C**), and SDM (**D**).

The CAF groups exhibited a higher steatosis grade than the SD groups (Figure 2, Figure 3 and Figure 4). In the SD groups, PVE and metformin lowered steatosis, with metformin reducing steatosis more than PVE, and both doses of PVE showed no differences. In the CAF groups, PVE lowered steatosis, but metformin only slightly reduced steatosis, and a high dose of PVE increased steatosis slightly more than a low dose. Table 7 presents the mean adipocyte area (μm^2^) across eight experimental groups expressed as means ± standard deviation. The data suggest that the “CAF” condition led to significant adipocyte hypertrophy. Both low and high doses of PVE treatments, as well as metformin, effectively reduced adipocyte size in the “CAF” model. Furthermore, the high dose of PVE treatment demonstrated an ability to reduce adipocyte size even under standard dietary conditions. These findings underscore the potential therapeutic effects of the tested interventions on adipocyte morphology.

### 3.8. Tissue Index

The tissue index of rat organs was calculated as the ratio of organ weight to final weight, and the result is shown in Table 8. Neither the SD nor CAF groups showed significant differences in the liver tissue index when compared under the same oral administration (*p* > 0.05) except the administration with the vehicle, in which the CAF led to a significantly higher liver tissue index than the SD group (*p* < 0.05). Among the SD groups, metformin exhibited a significantly higher liver tissue index than the vehicle control (*p* < 0.05), while the low- and high-PVE administration led to no significant difference in the liver tissue index compared to the vehicle control (*p* > 0.05); meanwhile, among the CAF groups, the low-PVE, high-PVE, and metformin treatments did not lead to a significantly different liver tissue index compared to the vehicle control (*p* > 0.05).

Oral administration with vehicles, SD, and CAF did not lead to a significant difference in the heart tissue index (*p* > 0.05). Meanwhile, oral administration with low PVE, high PVE, and metformin in the CAF group exhibited a significantly lower heart tissue index than in the SD group receiving the same oral administration (*p* < 0.05). Among the SD groups, low and high PVE led to a significantly lower heart tissue index than the vehicle control (*p* < 0.05), while metformin did not lead to a significantly different heart tissue index compared to the vehicle control (*p* > 0.05). Meanwhile, among the CAF groups, neither low PVE, high PVE, or metformin led to a significantly different heart tissue index compared to the vehicle control (*p* > 0.05).

The CAF groups exhibited a significantly lower kidney tissue index than the SD groups when compared under the same oral administration (*p* < 0.05). Neither the SD nor CAF groups orally administrated with low and high doses of PVE and metformin showed a significantly different kidney tissue index compared to the vehicle control (*p* > 0.05). In addition, the CAF groups exhibited a significantly higher VAT index than the SD groups when compared under the same oral administration (*p* < 0.05). In both the SD and CAF groups orally administrated with low and high doses of PVE and metformin, there was no significant difference in the VAT index compared to the vehicle control (*p* > 0.05).

**Figure 5:**
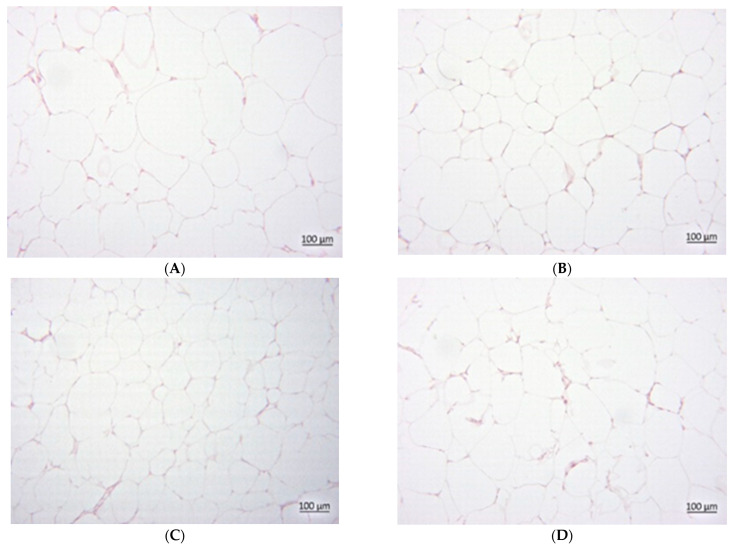
eWAT of CAFV (**A**), CAFLP (**B**), CAFHO (**C**), and CAFM (**D**).

In the CAF groups, oral administration with the vehicle and administration of a low dose of PVE led to a higher eWAT index than the SD groups under the same treatment (*p* < 0.05). In the SD and CAF groups, oral administration with metformin and a high dose of PVE led to no significant difference in the eWAT index than other groups under the same treatment (*p* > 0.05). Among the SD groups, metformin and a high dose of PVE led to a significantly higher eWAT index than the vehicle control (*p* < 0.05), while a low dose of PVE did not lead to a significantly different eWAT index compared to the vehicle control (*p* > 0.05). Meanwhile, among the CAF groups, low and high doses of PVE and metformin did not lead to a significantly different eWAT index compared to the vehicle control (*p* > 0.05). The CAF groups exhibited a significantly lower soleus muscle tissue index than the SD groups under the same treatment (*p* < 0.05). Neither the SD nor CAF groups orally administrated with low or high doses of PVE or metformin exhibited a significantly different soleus muscle tissue index compared to the vehicle control (*p* > 0.05) (Figure 5).

## 4. Discussion

This study investigated the impact of *Phaseolus vulgaris* (PVE) on body weight regulation in a cafeteria diet (CAF)-induced overweight rat model. Male Wistar rats (*n* = 48) were initially divided into two groups (*n* = 24 per group): a control group maintained on a standard diet and a CAF group designed to induce overweight over a 16-week period. As anticipated, the CAF group exhibited a significantly increased body weight gain and energy intake compared to the control group. Subsequently, both the control and CAF groups were further subdivided into four treatment subgroups *(n* = 6 per subgroup), receiving a daily oral administration of either the vehicle, a low dose of PVE (200 mg/kg), a high dose of PVE (300 mg/kg), or metformin (200 mg/kg) for an additional 6 weeks. The results demonstrated that the administration of a high dose of PVE and metformin significantly attenuated body weight gain in the CAF-induced overweight rats. These findings suggest PVE’s potential role in mitigating weight gain associated with a palatable, high-energy diet.

The cafeteria diet (CAF) is used in animal models to study obesity and its related metabolic disorders, including diabetes mellitus. This diet typically consists of a variety of highly palatable, energy-dense foods that mimic the “Western diet” commonly consumed by humans. These foods are often high in fat, sugar, and processed ingredients.

The consumption of a cafeteria diet (CAF) triggers a cascade of metabolic dysfunctions, initially manifesting as hyperglycemia and compensatory hyperinsulinemia due to elevated blood glucose levels [20,21]. This is followed by the development of insulin resistance, characterized by a reduced cellular response to insulin and consequently impaired glucose uptake [20,21]. Furthermore, CAF intake leads to dyslipidemia, which is marked by abnormal lipid profiles, including increased triglycerides and cholesterol concentrations. The diet also induces low-grade chronic inflammation in adipose tissue and the liver, which is a significant factor contributing to the observed insulin resistance [22]. Finally, CAF feeding promotes oxidative stress within adipose tissue, which further exacerbates inflammation and contributes to overall metabolic dysfunction [23].

The CAF increases obesity via an increasing body weight associated with energy intake [20]. In the study, the CAF groups had higher glycemia than the SD groups because the CAF led to hepatic steatosis and adiposity that involved weight gain, inflammation, insulin resistance, and obesity related with macrophage infiltration [20].

This study investigated the efficacy of *Phaseolus vulgaris* extract (PVE) in mitigating the detrimental effects of a cafeteria diet (CAF) on obese rats. Our findings revealed that PVE administration significantly reduced body weight gain in CAF-fed rats, with lower concentrations exhibiting an effect comparable to that of metformin—a well-established agent in managing obesity and type 2 diabetes. The observed reduction in body weight gain by PVE is likely attributed to decreased food absorption, a mechanism previously reported by Tormo et al. (2004) [7].

Furthermore, PVE treatment demonstrably lowered glycemia in the CAF group, with lower concentrations also significantly reducing the area under the glucose tolerance curve (AUC), indicating improved glucose homeostasis. Beyond glucose regulation, PVE effectively lowered circulating levels of total cholesterol (TC), triglycerides (TGs), aspartate aminotransferase (AST), and alanine aminotransferase (ALT), suggesting a protective role against CAF-induced dyslipidemia and hepatic injury. In addition, the SDLP, SDHP, CAFV, CAFLP, and CAFHP groups showed lower AST levels compared to the SDV and SDM groups, with some of these differences being statistically significant (*p* < 0.05). The SDV and SDM groups exhibited the highest AST values and also the largest variability, particularly the SDM groups. The superscript letter “a” and “b” denote statistical significance when compared to their respective control groups, although the specific control for each annotation is not explicitly stated in the table itself.

Histological analyses further supported these findings, revealing that PVE administration attenuated hepatic steatosis and reduced adipocyte size in CAF-fed rats.

The beneficial effects of PVE can be primarily attributed to its αAI activity. As evidenced by [22], the αAIs present in *Phaseolus vulgaris* can suppress pancreatic α-amylase, leading to diminished starch digestion and absorption in the intestine. This reduced carbohydrate assimilation likely underlies the observed lower body weight gain, improved glycemic control, and the subsequent reduction in hepatic steatosis and inflammation. In conclusion, our data suggest that *Phaseolus vulgaris* extract presents a promising therapeutic strategy for combating obesity and associated metabolic complications arising from the consumption of a high-energy, palatable diet.

Analysis of the tissues indices revealed that neither *Phaseolus vulgaris* extract (PVE) nor metformin administration exerted a significant effect on the relative weights of the heart, kidney, and soleus muscle. As is consistent with the induction of obesity, the cafeteria diet (CAF) led to a reduction in the tissue indices of these lean tissues (heart, kidney, and soleus) while conversely increasing the tissue indices of visceral adipose tissue (VAT) and epididymal white adipose tissue (eWAT). The lower tissue indices observed in the heart, kidney, and soleus of CAF-fed rats are likely attributable to the lower density of adipocytes compared to lean tissue. The increased adiposity resulting from the CAF leads to fat infiltration and the potential replacement of denser lean tissue with less dense fat cells, thus lowering the overall tissue index. Conversely, the elevated tissue indices of VAT and eWAT in the CAF group are expected, as these represent the primary sites of triacylglycerol (TG) storage in response to an increased energy intake [24].

Considering the acute effects of PVE, previous research has indicated its potential to reduce ghrelin levels and diminish the desire to eat alongside its αAI activity, which can slow gastric emptying [12]. While these acute effects on appetite regulation and gastric motility may have contributed to the observed long-term reductions in body weight gain, they did not translate into significant alterations in the relative weights of the examined lean tissues in this chronic study. This suggests that the primary impact of PVE and metformin on body composition in this model may be mediated through mechanisms affecting overall adiposity rather than directly influencing the relative mass of individual lean organs. Further investigation into the specific mechanisms underlying the differential effects of these treatments on lean versus adipose tissue mass is warranted.

This study, while acknowledging the limitations of the cafeteria-diet-induced obese rat model in fully replicating human obesity’s complexity, affirms its utility for preclinical metabolic research. The authors recognize and statistically addressed intra- and intergroup variability inherent in in vivo studies, maintaining that the observed trends are valid. However, they concede the lack of rigorous extract standardization for probiotics/metformin as a limitation, committing to improved characterization in future work. Despite the modest magnitude of some metabolic effects, the statistically significant improvements in markers like AST, ALT, and BUN suggest a positive physiological impact, warranting further investigation with optimized interventions.

## 5. Conclusions

*Phaseolus vulgaris* extract (PVE) demonstrates significant potential in mitigating cafeteria diet (CAF)-induced obesity and metabolic dysfunction in rats. Low-dose PVE effectively reduced body weight gain and improved glucose tolerance in obese rats, likely through α-amylase inhibition, leading to reduced carbohydrate absorption and subsequent lower food intakes. Furthermore, PVE exhibited beneficial effects on lipid profiles and may offer protection against hepatic steatosis. These in vivo findings highlight the therapeutic promise of *P. vulgaris* extract for managing obesity associated with high-energy diets, warranting further investigation into optimal dosage and clinical translation.

## Figures and Tables

**Table 1:** Initial and final body weights and body weight gain in male Wistar rats receiving SDV, SDLP, SDHP, SDM, CAFV, CAFLP, CAFHP, and CAFM.

Group	Initial Body Weight (g)	Final Body Weight (g)	Body Weight Gain (g)
SDV	319 ± 40	602 ± 64 ^a^	283 ± 35
SDLP	331 ± 33	578 ± 56 ^b^	247 ± 25
SDHP	318 ± 12	548 ± 29 ^b^	230 ± 28
SDM	320 ± 30	597 ± 49 ^a^	277 ± 25
CAFV	319 ± 32	810 ± 127 * ^,a^	491 ± 110 *^,a^
CAFLP	338 ± 33	722 ± 43 *^,ab^	384 ± 45 *^,b^
CAFHP	320 ± 39	776 ± 128 *^,a^	456 ± 96 *^,a^
CAFM	323 ± 14	678 ± 70 *^,bc^	356 ± 61 *^,b^

Values are expressed as means ± S.D. * Significant difference between SD and CAF groups with the same treatment (*p* < 0.05). ^abc^ Significant difference between vehicle and treatment groups (*p* < 0.05).

**Table 2:** Food consumption of male Wistar rats receiving SDV, SDLP, SDHP, SDM, CAFV, CAFLP, CAFHP, and CAFM.

Group	Week 0 (g)	Week 1 (g)	Week 2 (g)	Week 3 (g)	Week 4 (g)	Week 5 (g)	Week 6 (g)
SDV	149 ± 9 ^a^	160 ± 6 ^ab^	152 ± 3 ^b^	149 ± 7 ^ab^	164 ± 6 ^a^	161 ± 7 ^ab^	147 ± 11 ^b^
SDLP	137 ± 16 ^b^	149 ± 11 ^b^	150 ± 12 ^bc^	155 ± 12 ^ab^	168 ± 7 ^a^	166 ± 7 ^a^	150 ± 4 ^a^
SDHP	144 ± 14 ^a^	149 ± 6 ^b^	146 ± 14 ^c^	143 ± 15 ^b^	152 ± 18 ^ab^	155 ± 13 ^b^	149 ± 20 ^a^
SDM	149 ± 8 ^a^	166 ± 4 ^a^	167 ± 15 ^a^	159 ± 14 ^a^	163 ± 13 ^a^	167 ± 14 ^a^	152 ± 19 ^a^
CAFV	129 ± 20 *^,a^	136 ± 19 *^,a^	151 ± 13 ^a^	147 ± 15 ^a^	142 ± 21 *^,a^	147 ± 22 ^a^	157 ± 23 ^a^
CAFLP	112 ± 5 *^,b^	119 ± 8 *^,b^	135 ± 7 *^,c^	127 ± 11 *^,c^	110 ± 15 *^,b^	132 ± 5 *^,b^	138 ± 6 *^,bc^
CAFHP	118 ± 7 *^,b^	127 ± 7 *^,ab^	147 ± 8 ^ab^	141 ± 6 ^b^	140 ± 9 ^a^	142 ± 8 ^ab^	149 ± 6 ^b^
CAFM	101 ± 3 *^,c^	117 ± 13 *^,b^	137 ± 11 *^,bc^	129 ± 12 *^,bc^	119 ± 9 *^,b^	120 ± 11 *^,c^	130 ± 4 *^,c^

Values are expressed as means ± S.D. * Significant difference between SD and CAF groups with the same treatment (*p* < 0.05). ^abc^ Significant difference between vehicle and treatment groups (*p* < 0.05).

**Table 3:** Energy intake of male Wistar rats receiving SDV, SDLP, SDHP, SDM, CAFV, CAFLP, CAFHP, and CAFM.

Group	Week 0 (kJ)	Week 1 (kJ)	Week 2 (kJ)	Week 3 (kJ)	Week 4 (kJ)	Week 5 (kJ)	Week 6 (kJ)
SDV	477 ± 30	510 ± 19	489 ± 10 ^ab^	476 ± 22	525 ± 19	517 ± 23	472 ± 35
SDLP	437 ± 52	478 ± 36	480 ± 40 ^ab^	496 ± 39	538 ± 24	531 ± 22	479 ± 14
SDHP	459 ± 43	478 ± 18	469 ± 44 ^b^	458 ± 49	487 ± 58	495 ± 41	476 ± 64
SDM	475 ± 24	530 ± 14	535 ± 49 ^a^	510 ± 46	523 ± 42	536 ± 44	487 ± 61
CAFV	651 ± 84 *	692 ± 87 *^,a^	770 ± 45 *^,a^	734 ± 57 *^,a^	703 ± 79 *^,a^	782 ± 94 *	837 ± 102 *
CAFLP	588 ± 22 *	664 ± 30 *^,ab^	701 ± 44 ^b^	667 ± 51 *^,b^	639 ± 37 *^,b^	725 ± 22 *	770 ± 40 *
CAFHP	608 ± 38 *	653 ± 40 *^,ab^	764 ± 60 *^,a^	740 ± 31 *^,a^	700 ± 49 *^,a^	735 ± 38 *	793 ± 38 *
CAFM	538 ± 17 *	627 ± 65 *^,b^	757 ± 52 *^,a^	712 ± 43 *^,ab^	652 ± 42 *^,ab^	729 ± 51 *	779 ± 19 *

Values are expressed as means ± S.D. * Significant difference between SD and CAF groups with the same treatment (*p* < 0.05). ^ab^ Significant difference between vehicle and treatment groups (*p* < 0.05).

**Table 4:** Glucose tolerance test (OGTT) of male Wistar rats receiving SDV, SDLP, SDHP, SDM, CAFV, CAFLP, CAFHP, and CAFM.

Group	0 Min	30 Min	60 Min	90 Min	120 Min
SDV	105 ± 11	145 ± 24	147 ± 19	141 ± 10 ^b^	134 ± 7
SDLP	103 ± 5	136 ± 16	145 ± 9	147 ± 9 ^a^	135 ± 15
SDHP	106 ± 10	140 ± 9	150 ± 6	151 ± 9 ^a^	128 ± 9
SDM	110 ± 8	147 ± 19	154 ± 12	151 ± 21 ^a^	137 ± 6
CAFV	120 ± 7 *	176 ± 26 *^,a^	185 ± 33 *^,a^	194 ± 37 *^,a^	183 ± 30 *^,a^
CAFLP	110 ± 14	157 ± 17 *^,b^	159 ± 9 *^,b^	161 ± 11 *^,b^	154 ± 11 *^,b^
CAFHP	114 ± 11	181 ± 29 *^,a^	188 ± 33 *^,a^	188 ± 35 *^,a^	170 ± 41 *^,a^
CAFM	119 ± 16	153 ± 27 ^b^	175 ± 22 *^,ab^	175 ± 23 *^,ab^	154 ± 21 *^,b^

Values are expressed as means ± S.D. * Significant difference between SD and CAF groups with the same treatment (*p* < 0.05). ^ab^ Significant difference between vehicle and treatment groups (*p* < 0.05).

**Table 5:** Area under the curve (AUC) for male Wistar rats receiving SDV, SDLP, SDHP, SDM, CAFV, CAFLP, CAFHP, and CAFM.

Group	AUC
SDV	1243 ± 689
SDLP	1075 ± 296
SDHP	1165 ± 328
SDM	1208 ± 209
CAFV	1550 ± 684 *^,a^
CAFLP	1105 ± 303 ^b^
CAFHP	1495 ± 514 *^,a^
CAFM	1525 ± 509 *^,a^

Values are expressed as means ± S.D. * Significant difference between SD and CAF groups with the same treatment (*p* < 0.05). ^ab^ Significant difference between vehicle and treatment groups (*p* < 0.05).

**Table 6:** Blood chemistry for male Wistar rats receiving SDV, SDLP, SDHP, SDM, CAFV, CAFLP, CAFHP, and CAFM.

Group	TC	TG	LDL	HDL	AST	ALT	BUN	Creatinine
SDV	81 ± 9	217 ± 51	<30	58 ± 7	287 ± 91 ^a^	36 ± 5 ^a^	15 ± 1 ^a^	0.5 ± 0 ^ab^
SDLP	64 ± 11	144 ± 39	<30	49 ± 8	102 ± 31 ^b^	22 ± 4 ^b^	12 ± 2 ^b^	0.4 ± 0.1 ^b^
SDHP	68 ± 2	200 ± 46	<30	51 ± 3	169 ± 35 ^b^	24 ± 5 ^b^	13 ± 3 ^ab^	0.5 ± 0.1 ^a^
SDM	74 ± 17	204 ± 60	<30	55 ± 10	275 ± 164 ^a^	32 ± 11 ^ab^	14 ± 2 ^ab^	0.5 ± 0.1 ^ab^
CAFV	88 ± 36	207 ± 83	<30	62 ± 23	164 ± 50 *^,b^	27 ± 15 ^a^	7 ± 2 *	0.4 ± 0.1 ^b^
CAFLP	76 ± 22	213 ± 52 *	<30	53 ± 16	113 ± 49 ^b^	23 ± 5 ^ab^	8 ± 2 *	0.5 ± 0.1 *^,a^
CAFHP	76 ± 13	176 ± 47	<30	56 ± 8	124 ± 50 ^b^	31 ± 9 ^a^	8 ± 3 *	0.4 ± 0.1 *^,b^
CAFM	72 ± 15	186 ± 47	<30	53 ± 11	198 ± 98 ^a^	20 ± 7 *^,b^	9 ± 1 *	0.5 ± 0.0 ^a^

Values are expressed as means ± S.D. * Significant difference between SD and CAF groups with the same treatment (*p* < 0.05). ^ab^ Significant difference between vehicle and treatment groups (*p* < 0.05).

**Table 7:** Mean adipocyte area of adipose tissues for male Wistar rats receiving SDV, SDLP, SDHP, SDM, CAFV, CAFLP, CAFHP, and CAFM.

Group	Liver HV/N	Mean Adipocyte Area (µm^2^)
SDV	0/3	9823 ± 1479 ^a^
SDLP	0/3	9574 ± 1553 ^ab^
SDHP	0/3	8264 ± 440 ^b^
SDM	0/3	11,003 ± 3559 ^a^
CAFV	1/3	12,497 ± 2044 *^,a^
CAFLP	1/3	10,032 ± 1305 ^ab^
CAFHP	1/3	6159 ± 408 *^,b^
CAFM	1/3	7973 ± 816 ^b^

The results are expressed as number of rat with pathological finding/total number of rats. Liver: HV = Hepatic vacuolation. HD = Hepatic degeneration. N = Normal (very mild lesion). Values are expressed as means ± S.D. * Significant difference between SD and CAF groups with the same treatment (*p* < 0.05). ^ab^ Significant difference between vehicle and treatment groups (*p* < 0.05).

**Table 8:** Tissue index of male Wistar rats receiving SDV, SDLP, SDHP, SDM, CAFV, CAFLP, CAFHP, and CAFM.

Group	Liver	Heart	Kidney	VAT	eWAT	Soleus
SDV	2.66 ± 0.19	0.23 ± 0.01 ^a^	0.56 ± 0.01	9.93 ± 2.07	2.87 ± 0.25 ^b^	0.10 ± 0.01 ^a^
SDLP	2.71 ± 0.16	0.22 ± 0.01 ^b^	0.58 ± 0.01	9.88 ± 1.34	2.86 ± 0.45 ^b^	0.10 ± 0.02 ^a^
SDHP	2.79 ± 0.19	0.21 ± 0.02 ^b^	0.56 ± 0.02	10.84 ± 1.52	3.36 ± 0.42 ^a^	0.10 ± 0.01 ^a^
SDM	3.09 ± 0.52	0.23 ± 0.02 ^a^	0.61 ± 0.02	11.82 ± 2.40	3.42 ± 0.40 ^a^	0.09 ± 0.01 ^b^
CAFV	3.15 ± 0.48 *	0.19 ± 0.06 ^b^	0.46 ± 0.06 *	16.31 ± 4.88 *	3.57 ± 0.64 *^,b^	0.07 ± 0.02 *^,b^
CAFLP	2.79 ± 0.21	0.19 ± 0.01 *^,b^	0.44 ± 0.01 *	16.48 ± 1.76 *	3.86 ± 0.74 *^,a^	0.07 ± 0.01 *^,b^
CAFHP	2.99 ± 0.62	0.19 ± 0.02 *^,b^	0.45 ± 0.02 *	18.86 ± 2.79 *	3.97 ± 0.89 ^a^	0.07 ± 0.01 *^,b^
CAFM	2.90 ± 0.21	0.21 ± 0.02 *^,a^	0.47 ± 0.02 *	17.02 ± 2.51 *	4.20 ± 1.01 ^a^	0.08 ± 0.01 *^,a^

Values are expressed as means ± S.D. * Significant difference between SD and CAF groups receiving the same treatment (*p* < 0.05). ^ab^ Significant difference between vehicle and treatment groups (*p* < 0.05).

## Data Availability

The data presented in this study are available on request from the corresponding author. The data are not publicly available due to privacy restrictions.

## Supplementary Materials

The following supporting information can be downloaded at https://www.mdpi.com/article/10.3390/foods14122038/s1. Table S1: Cafeteria diet Formula for induction of obesity in male rats.
