# Supplementary material for: Evaluation of Phaseolus vulgaris Extract in a Rat Model of Cafeteria-Diet-Induced Obesity: Metabolic and Biochemical Effects
Source: Foods. 2025 Jun 9;14(12):2038. doi: 10.3390/foods14122038 (PMC12191728; doi:10.3390/foods14122038)
Supplement: Supplementary file 1 [file foods-14-02038-s001.zip › foods-3643693-supplementary.pdf]

Table S1. Cafeteria diet Formula for induction of obesity in male rats.

**Cafeteria Diet (CAF)**			**%**		
**Formula 1**		weight (g)	Carbohydrate	Protein	Fat
	Nutrients need		65	15	20
	Standard Diet (Chow)	60	26.88	14.4	2.7
	Nutrient for CAF		38.12	0.6	17.3
Salty	Lay (14 g)	25	16.07	1.79	7.14
	Lack nutrient		22.05	-1.19	10.16
Sweety	Butter cake Euro (40 g)	50	22.5	2.5	15
	Lack nutrient		-0.45	-3.69	-4.84
Total weight		135	**for 1 day/one rat**		

**Cafeteria Diet (CAF)**			**%**		
**Formula 2**		weight (g)	Carbohydrate	Protein	Fat
	Nutrients need		65	15	20
	Standard Diet (Chow)	60	26.88	14.4	2.7
	Nutrient for CAF		38.12	0.6	17.3
Salty			0	0	0
	Lack		38.12	0.6	17.3
Sweety	Cheese cookie (RITZ)	68	37.8	5.0388	20.1416
	Lack		0.3	-4.4388	-2.8416
Total weight		128	**for 1 day/one rat**		

**Cafeteria Diet (CAF)**			**%**		
**Formula 3**		weight (g)	Carbohydrate	Protein	Fat
	Nutrients need		65	15	20
	Standard Diet (Chow)	60	26.88	14.4	2.7
	Nutrient for CAF		38.12	0.6	17.3
Salty	Smoke sausage (CP)	20	0	1.272	5
	Lack		38.12	-0.672	12.3
Sweety	Imperial cookie vanilla ring	65	42.7	4.0625	16.25
	Lack		-4.5	-4.7345	-3.95
Total weight		145	**for 1 day/one rat**		
